# Effects of Morbid Obesity and Metabolic Syndrome on the Composition of Circulating Immune Subsets

**DOI:** 10.3389/fimmu.2021.675018

**Published:** 2021-07-20

**Authors:** Leontine H. Wijngaarden, Erwin van der Harst, René A. Klaassen, Martin Dunkelgrun, T Martijn Kuijper, Mariska Klepper, Gisela Ambagtsheer, Jan N. M. IJzermans, Ron W. F. de Bruin, Nicolle H. R. Litjens

**Affiliations:** ^1^ Department of Surgery, Maasstad Hospital, Rotterdam, Netherlands; ^2^ Department of Surgery, Erasmus MC, University Medical Center Rotterdam, Rotterdam, Netherlands; ^3^ Department of Surgery, Franciscus Gasthuis and Vlietland, Rotterdam, Netherlands; ^4^ Maasstad Academy, Maasstad Hospital, Rotterdam, Netherlands; ^5^ Department of Internal Medicine, Section Nephrology and Transplantation, Erasmus MC, University Medical Center Rotterdam, Rotterdam, Netherlands

**Keywords:** T cells, B cells, NK cells, metabolic syndrome, morbid obesity, monocytes, metaflammation

## Abstract

Morbid obesity is characterized by chronic, low-grade inflammation, which is associated with ‘inflamm-aging’. The presence of metabolic syndrome (MetS) might accelerate this phenomenon of metaflammation. In this study, we assessed the effects of morbid obesity and MetS on the composition of a broad spectrum of immune cells present within the circulation. A total of 117 morbidly obese patients (MOP) without MetS (MetS-), 127 MOP with MetS (MetS+) and 55 lean controls (LC) were included in this study. Absolute numbers of T cell, B cell, NK cell and monocyte subsets were assessed within peripheral blood using flow cytometry. Both absolute cell numbers and proportion of cells were evaluated correcting for covariates age, body mass index and cytomegalovirus serostatus. Although the absolute number of circulating CD4+ T cells was increased in the MetS+ group, the CD4+ T cell composition was not influenced by MetS. The CD8+ T cell and B cell compartment contained more differentiated cells in the MOP, but was not affected by MetS. Even though the absolute numbers of NK cells and monocytes were increased in the MOP as compared to LC, there was no difference in proportions of NK and monocyte subsets between the three study groups. In conclusion, although absolute numbers of CD4+ and CD8+ T cells, B cells, NK cells and monocytes are increased in MOP, obesity-induced effects of the composition of the immune system are confined to a more differentiated phenotype of CD8+ T cells and B cells. These results were not affected by MetS.

## Introduction

Morbid obesity is characterized by a state of chronic, low-grade inflammation (1). This systemic inflammation, also called metaflammation, is caused by the high number of adipocytes in the white adipose tissue. Especially metabolic overload leads to adipocyte dysfunction (2). This secretes pro-inflammatory cytokines such as tumor necrosis factor α (TNF-α), interleukins (IL-) 2 and 6, and C-reactive protein (CRP) (3). Metaflammation is associated with accelerated aging, referred to as ‘inflamm-aging’ (1, 4). This phenomenon is especially described in morbidly obese individuals with metabolic syndrome (MetS), which is characterized by dyslipidemia, dysglycemia, an elevated blood pressure and an increased abdominal waist circumference (5, 6). Clinical consequences of metaflammation include a decreased vaccination efficacy, an increased risk for developing cardiovascular diseases and type 2 diabetes (T2D), and an increased mortality rate (4, 7, 8).

Aging affects different components of the adaptive as well as the innate immune system, the former being more extensively studied. In the aging population, there is a phenotypic shift of the lymphoid to the myeloid lineage (9). This eventually contributes to immune dysfunction in the older population. For the adaptive immune system, thymic involution caused by aging leads to a decrease in circulating recent thymic emigrants (RTEs), which can be identified by CD31-expression within naive circulating T cells (10). Furthermore, there is a shift from CD45RO^-^CCR7^+^ naïve T cells to CD45RO^+^ memory and CD45RO^-^CCR7^-^ terminally differentiated effector memory (EMRA) T cells (1, 11). Additionally, loss of CD28 on the membrane of T cells leads to an increase in advanced differentiated CD28^null^ T cells in the aging population. A comparable shift is seen in B cell subpopulations, resulting in a decrease of CD24^high^ transitional and CD27^-^ naïve B cells, and an increase in a more differentiated phenotype of B cell subsets, including CD27^+^ switched and non-switched B cells, CD27^-^IgD^-^ double negative B cells and an increase in CD27^high^ plasma blasts (12, 13). For the innate immune system, an age-related phenotypic change of NK cells and monocytes is described. Whereas the immunomodulatory CD56^bright^ NK cells do not seem to be influenced by aging, an increase in mature, cytotoxic CD56^dim^ NK cells has been described (14, 15). Additionally, in aging there is a shift from the pro-inflammatory classical CD14^+^CD16^-^ monocytes to the anti-inflammatory non-classical CD14^dim^CD16^+^ monocytes (16).

Several studies have investigated the effect of obesity on immunosenescence, and found a phenotypically aged profile among morbidly obese individuals (17–23). However, most studies investigated only one or two specific immune cell subsets instead of a broad spectrum of circulating immune cells. Additionally, study populations were relatively small and studies were performed in specifically chosen study populations, which do not reflect the general population at the outpatient clinic. In addition, not all studies included a lean healthy control group. To our knowledge, we were the first to study the influence of MetS and corrected for confounders such as cytomegalovirus (CMV) seropositivity (24). Undoubtedly, correction for CMV status should be performed as CMV seropositivity is associated with an increase in differentiated memory T cells, and thus an aged immune profile (25). Therefore, CMV seropositivity can influence the outcomes of our research question.

In summary, the aim of this study was to assess the effects of morbid obesity and MetS on phenotypical changes of the adaptive as well as the innate immune system in a large cohort of morbidly obese patients as compared to lean subjects, with correction for CMV status.

## Materials and Methods

### Patient Selection

Morbidly obese patients who were scheduled for laparoscopic Roux-en-Y gastric bypass (LRYGB) in the Maasstad Hospital and Sint Franciscus Gasthuis & Vlietland, Rotterdam, the Netherlands between June 2018 and October 2019 were invited to participate in this prospective cohort study. To be eligible for LRYGB, patients had to fulfill the criteria for bariatric surgery of the International Federation for the Surgery of Obesity and Metabolic Disorders (IFSO) (26). Exclusion criteria were lack of basic understanding of the Dutch or English language, or previous bariatric surgery in the medical history. In order to reflect the general bariatric population of the outpatient clinic, there were no exclusion criteria based on comorbidities, medication use prior to bariatric surgery, use of supplements or dietary intake.

Between December 2018 and April 2019, blood donors at the Sanquin blood bank were invited to participate in this study as lean, healthy controls. Controls with a Body Mass Index (BMI) ≥ 30 kg/m^2^ or with the presence of MetS were excluded from this study. BMI was calculated using a person’s height and weight with the following formula: . Lean controls were included to analyze the effect of morbid obesity (with a distinction between morbidly obese patients with *versus* without MetS) on the immune system, as lean controls do not have an accumulation of white adipose tissue leading to metaflammation and thus affect the phenotype of the immune system.

A sample size calculation was performed prior to the start of this study. According to this sample size calculation, the aim was to include 125 patients in each morbidly obese groups and 60 lean controls. The local medical ethical committee (MEC), being the Medical research Ethics Committees United, approved the study (MEC number: MEC-2018-06). All participants of this study gave written informed consent. This study was conducted in accordance with the Declaration of Helsinki and the Declaration of Istanbul and in compliance with the International Conference on Harmonization/Good Clinical Practice regulations.

### Metabolic Syndrome

Patients were clinically diagnosed with MetS if they had the presence of three or more of the following risk factors (6, 27):

- An increased waist circumference (≥ 102 cm in males, ≥ 88 cm in females)
- Elevated triglycerides (≥ 150 mg/dL or 1.7 mmol/L) or drug treatment for elevated triglycerides
- Reduced HDL cholesterol (<40 mg/dL or 1.0 mmol/L in males, <50 mg/dL or 1.3 mmol/L in females) or drug treatment for reduced HDL cholesterol
- Elevated blood pressure (systolic ≥ 130 mmHg and/or diastolic ≥ 85 mmHg) or antihypertensive drug treatment
- Elevated fasting glucose (≥100 mg/dL) or antidiabetic drug treatment

Blood pressure was measured with the patient sitting in an upright position with the back supported for at least five minutes and the arm supported at the level of the heart, using an automatic sphygmomanometer (Welch Allyn, Hillrom Holdings, Inc., Chicago, IL, USA). The triglycerides, HDL cholesterol and fasting glucose were measured directly in serum obtained by vena puncture.

### Blood Collection

In the morbidly obese population, venous blood samples were obtained at the clinic on the day of surgery, prior to the surgical intervention. Blood samples were collected in either two 10.0 mL or four 6.0 mL Lithium-Heparin tubes (BD, Franklin States NJ, USA). The blood samples were stored at room temperature and were analyzed within 8 hours after blood collection.

In the lean control population, blood was collected in two 10.0 mL Lithium-Heparin tubes prior to blood donation. The blood samples were stored at room temperature and were analyzed within 8 hours after blood collection.

### CMV Serology

The diagnostic department of Virology assessed the CMV serology of all included participants. This was performed by determining the presence of plasma IgG antibodies to CMV with an enzyme immune assay (Biomerieux, VIDAS, Lyon, France). An outcome of ≥ 6 arbitrary units/mL (AU/mL) was considered as positive.

### Immune Cell Phenotyping

Whole blood stainings were performed using fluorescently-labelled antibodies to identify and determine frequencies as well as absolute numbers of the different circulating immune cells and their differentiation status. **Supplementary Table 1** lists the circulating immune cells we measured and the markers used for their identification.

Briefly, Trucount tubes (BD Pharmingen, Erebodegem, Belgium) containing a fixed number of beads were used to determine absolute numbers of leukocytes. Whole blood was incubated for 15 minutes at room temperature with blue violet 510 (BV510)-labeled anti-CD3, Pacific Blue (PacBlue)-labeled anti-CD4, fluorescein isothiocyanate (FITC)-labeled anti-CD8, phycoerythrin/cyanine7 (PE-Cy7)-labeled anti-CD19, allophycocyanin (APC)-labeled anti-CD45, PE-labeled anti-CD16, peridin chlorophyll protein (PerCP)-Cy7-labeled anti-CD56 (Biolegend Europe B.V. Uithoorn, Netherlands), and APC-H7-labeled anti-CD14 (BD). Subsequently, cells were lysed with Pharm Lyse solution (BD, diluted 10x with Milli-Q water) for 15 minutes. Afterwards, cells numbers were determined on a FACSCanto II equipped with 3 lasers (Blue laser harboring 4 detectors, red laser harboring 2 detectors and violet laser harboring 2 detectors; BD Biosciences, Erembodegem, Belgium) using FACSDiva software version 8 (BD).

An additional staining was performed to identify the different T cell subsets within CD4+ and CD8+ T cells. Whole blood staining using BV510-labeled anti-CD3, PacBlue-labeled anti-CD4, APC-Cy7-labeled anti-CD8, APC-labeled anti-CD45RO, PE-Cy7-labeled anti-CCR7, PE-labeled anti-CD31 (Biolegend), and PerCP-Cy5.5-labeled anti-CD28 (BD) were added to whole blood. The different B cell subsets were determined in a separate staining, using BV510-labeled anti-CD19, PE-Cy7-labeled anti-CD27, APC-Cy7-labeled anti-IgD, APC-labeled anti-CD24 (Biolegend), and BV421-labeled anti-CD38 (BD). Both staining tubes were incubated for 15 minutes at room temperature. Subsequently, cells were lysed for 10 minutes at room temperature using BD FACS lysing solution (BD), centrifuged for 5 minutes at 2000 RPM and washed twice using FACS flow solution (BD). Afterwards, cells were measured on a FACSCanto II (BD) using FACSDiva software version 8 (BD).

The cells were analyzed using Kaluza Analysis Software version 2.1 (Beckman Coulter, Indianapolis, USA). A typical example of the flow cytometric analysis and gating strategy used is depicted in **Supplementary Figure 1**.

### Statistical Analysis

A sample size calculation was performed, by which we aimed to include 125 patients in both morbidly obese patients groups and 60 patients in the lean control group.

Baseline characteristics are reported using simple descriptive statistics. Comparisons between groups were performed using Pearson’s chi –square test for categorical data and Mann-Whitney U test for continuous data. A mixed negative binomial regression model with a random intercept for each subject was used for the statistical analysis of cell counts and cell subset composition. A Dirichlet multinomial mixed model was used for statistical analysis of cell subset composition in percentages (28). Additionally, the effects of covariates were investigated by including interactions for cell type and covariates age, BMI and CMV (yes/no). BMI was centered at the medians of the respective groups to allow for selective adjustment of within-group differences only. Thus, effects due to between-group differences in BMI were captured by the indicator variables for groups. Age was centered at the overall median to allow for easier interpretation of coefficients. The dispersion parameter was modeled as a function of the expected mean. Significance of differences in cell counts was tested by multivariate Wald tests in a sequential fashion. First, an overall test was done to assess differences for any cell type between groups. If significant, separate follow-up tests were performed for each cell type. Statistical analysis was performed using Stata version 14.2 (StataCorp, Texas, USA) and R version 3.6.1 (R Core Team, R Foundation for Statistical Computing, Vienna, Austria). A two-sided *P*-value <0.05 was used to indicate statistical significance.

## Results

### Baseline Characteristics

The study population consisted of 55 lean controls (LC), 117 morbidly obese patients without metabolic syndrome (MetS-) and 127 morbidly obese patients with metabolic syndrome (MetS+). The immune status of 60 lean controls were analyzed, however, five lean controls were excluded from this study as they had a BMI > 30 kg/m^2^.

The LC were significant younger than the MetS- and MetS+ groups (*P*<0.001). Baseline characteristics are shown in **Table 1**. The BMI of LC was significantly lower than that of morbidly obese patients (*P*<0.001), however, there was no significant difference in BMI between the MetS- and MetS+ groups (*P*=0.095). There were no significant differences in CMV seropositivity between the three study groups. Four MetS+ patients had a BMI <35 kg/m^2^ on the day of surgery. These patients all had a BMI >35 kg/m^2^ on their first presentation at the outpatient clinic. As a result of participation in an intervention program, consisting of psychological, dietetic and physiotherapeutic support to adjust their life style prior to surgery, they had already lost weight preoperatively.

**Table 1:** Baseline characteristics.

	Lean controls (n= 55)	MetS- (n=117)	MetS+ (n= 127)	*P*-value
Age (median and range, in years)	31 [25;52]	42 [35;50]	50 [39;56]	<0.001
Weight (median and range, in kg)	75 [70;83]	115.5 [107.1;125.1]	119.5 [107.3;131.2]	<0.001
BMI (median and range, in kg/m^2^)	24.4 [22.5;26.8]	41.5 [40.0;43.4]	40.6 [37.4;43.6]	<0.001
BMI group (number, %)				<0.001
< 30 kg/m^2^	55 (100%)	0 (0%)	0 (0%)	
30 – 34.9 kg/m^2^	0 (0%)	0 (0%)	4 (3.2%)	
35 – 39.9 kg/m^2^	0 (0%)	29 (24.8%)	50 (39.4%)	
40 – 44.9 kg/m^2^	0 (0%)	66 (56.4%)	48 (37.8%)	
> 45 kg/m2	0 (0%)	22 (18.8%)	25 (19.7%)	
CMV seropositivity (number, %)	28 (50.9%)	61 (52.1%)	71 (55.9%)	0.767
Comorbidities (number, %)				
T2D		0 (0%)	43 (33.9%)	<0.001
HT		17 (14.5%)	68 (53.5%)	<0.001
HC		11 (9.4%)	35 (27.6%)	<0.001
OSAS		14 (12.0%)	27 (21.3%)	0.052

MetS-, morbidly obese patients without metabolic syndrome; Mets+, morbidly obese patients with metabolic syndrome; BMI, Body Mass Index; CMV, Cytomegalovirus; T2D, type 2 diabetes; HT, hypertension; HC, hypercholesterolemia; OSAS, obstructive sleep apnea syndrome.

### Morbid Obesity Induces an Aged CD8+ T Cell Compartment

**Figure 1:**
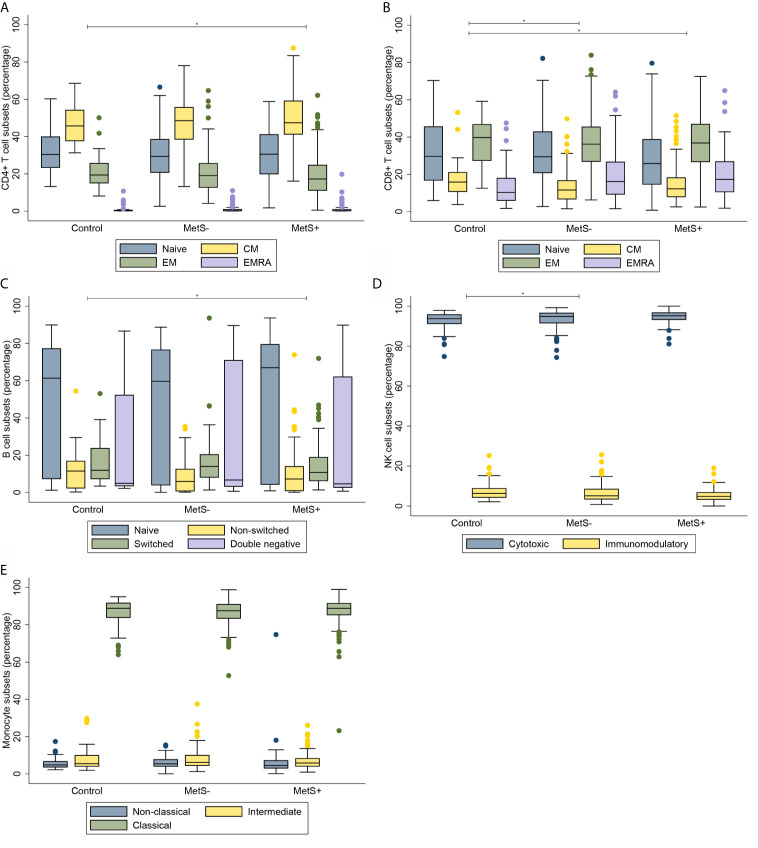
Composition of immune cell subsets in absolute cell counts. **(A)** CD4+ T cell subsets. **(B)** CD8+ T cell subsets. **(C)** B cell subsets. **(D)** NK cell subsets. **(E)** Monocyte subsets. MetS-, morbidly obese patients without metabolic syndrome; MetS+, morbidly obese patients with metabolic syndrome; CM, central memory; EM, effector memory; EMRA, terminally differentiated effector memory. *P < 0.05.

**Figure 2:**
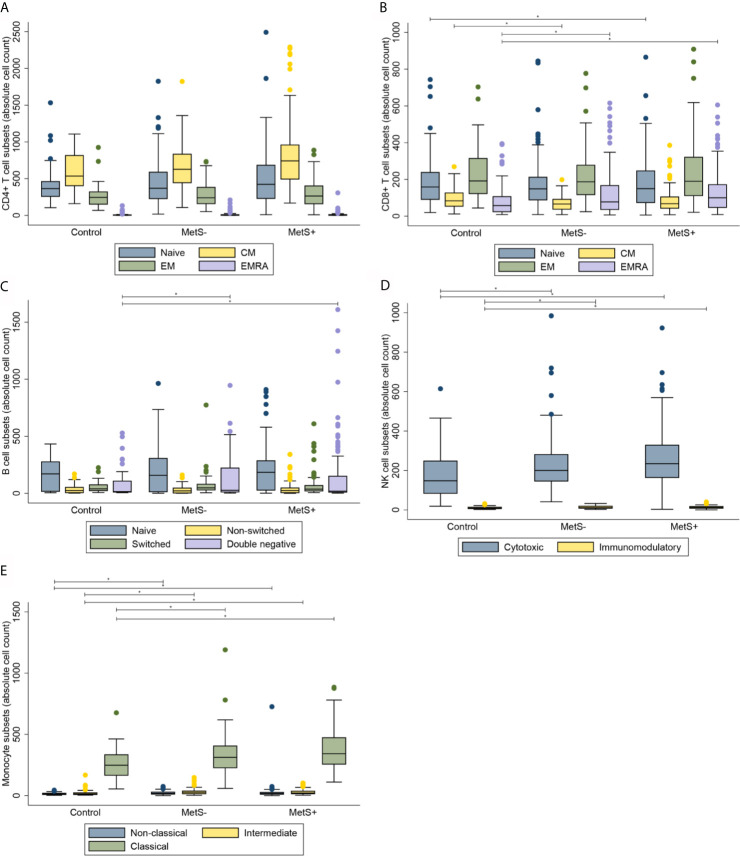
Composition of immune cell subsets in percentages. **(A)** CD4+ T cell subsets. **(B)** CD8+ T cell subsets. **(C)** B cell subsets. **(D)** NK cell subsets. **(E)** Monocyte subsets. MetS- = morbidly obese patients without metabolic syndrome; MetS+, morbidly obese patients with metabolic syndrome; CM, central memory; EM, effector memory; EMRA, terminally differentiated effector memory. *P < 0.05.

Morbidly obese patients had an increased number of circulating CD3+ T cells when compared to lean controls (*P*=0.010). The median [interquartile range] numbers of the circulating cells are shown in **Table 2A**. CMV seropositivity was significantly associated with higher overall CD3+ T cell counts (coefficient 0.110, *P*=0.014). The difference in number of circulating T cells was mainly caused by an increase in CD4+, but not CD8+, T cells in the MetS+ group. The increase in CD4+ T cells was not influenced by any of the confounders. Whereas the absolute number of circulating CD4+ T cells was increased in the MetS+ group, the presence of MetS was not associated with the composition of the CD4+ T cell subsets with respect to absolute numbers (*P*=0.156). In contrast to the CD4+ T cell subsets, the CD8+ T cell compartment contained more differentiated cells in the morbidly obese patients (*P*<0.001). This was reflected by an increase in CD8+ EMRA T cells in the morbidly obese patients as compared to lean controls (LC *vs* MetS- *P*=0.004, LC *vs* MetS+ *P*<0.001). MetS did not have an additional effect on CD8+ T cell differentiation (*P*=0.519), **Figures 1A, B**. There were no significant differences in CD4+ T cell subsets in terms of percentages (*P*=0.070), while CD8+ T cell subsets in terms of percentages were significantly different (*P*=0.033), as shown in **Figures 2A, B**. This was in both lean controls *versus* MetS- (*P*=0.043) and LC *versus* MetS+ (*P*=0.004).

**Table 2a:** Absolute numbers of the T cell subsets.

	Lean controls (n=55)	MetS- (n=177)	MetS+ (n=127)	*P*-value
CD3+	1975 [1448;2440]	2032 [1517;3451]	2255 [1596;2840]	0.010
CD4+	1278 [897;1539]	1286 [1023;1775]	1552 [1124;1990]	0.003
CD4+ naive CD31^+^	856 [675;1153]	968 [729;1314]	1070 [708;1393]	0.003
CD4+ naïve	365 [258;460]	369 [228;589]	422 [230;682]	0.156
CD4+ CM	535 [404;815]	628 [445;833]	742 [493;958]	0.156
CD4+ EM	246 [152;320]	240 [160;381]	263 [158;402]	0.156
CD4+ EMRA	3 [2;7]	3 [1;11]	3 [1;11]	0.156
CD4+ CD28^null^	10 [4;43]	6 [2;75]	8 [2;64]	0.234
CD8+	604 [340;772]	554 [389;770]	561 [398;816]	0.231
CD8+ CD31^naive^	574 [315;761]	547 [386;753]	540 [383;795]	0.237
CD8+ naïve	159 [91;238]	149 [88;212]	150 [74;247]	< 0.001
CD8+ CM	83 [54;127]	66 [37;91]	68 [43;105]	< 0.001
CD8+ EM	192 [123;314]	187 [117;277]	190 [112;320]	< 0.001
CD8+ EMRA	57 [24;106]	77 [36;166]	100 [47;172]	< 0.001
CD8+ CD28^null^	120 [59;216]	145 [76;286]	152 [79;329]	0.060

MetS-, morbidly obese patients without metabolic syndrome; MetS+, morbidly obese patients with metabolic syndrome; CM, central memory; EM, effector memory; EMRA, terminally differentiated effector memory.

All absolute numbers are presented as median [interquartile range]. P-values are after correction for covariates using a binomial mixed regression model.

### The CD4+/CD8+ Ratio Is Not Influenced by Metabolic Syndrome

Although there was a trend towards an increased CD4+/CD8+ ratio in the morbidly obese patients as compared to lean controls (2.06 [1.63;2.99] in LC, 2.31 [1.84;3.20] in MetS- and 2.57 [1.94;3.60] in MetS+), this was not significantly different after correction for the covariates (*P*=0.518).

### Morbidly Obese Patients Have Increased Numbers of Differentiated DN B Cells

Along with the absolute number of T cells, the absolute number of B cells was significantly different between the three study groups (*P*=0.001), as presented in **Table 2B**. Additionally, there was a significant difference in the absolute number of B cell composition (*P*=0.037). This was reflected by an increase of DN B cells in morbidly obese patients as compared to LC (LC *vs* MetS- *P*=0.011, LC *vs* MetS+ *P*<0.001), **Figure 1C**. Both age (*P*=0.010) and BMI (*P*=0.025) influenced this difference positively. Also, there was a significant increase in mature plasma blasts in the morbidly obese patients as compared to the lean controls (*P*<0.001). None of the confounders showed an additional effect on this increase in plasma blasts. Nevertheless, the composition of B cell subsets in percentages was not significantly different between the three study groups (*P*=0.152), **Figure 2C**.

**Table 2b:** Absolute numbers of the B cell subsets.

	Lean controls (n=55)	MetS- (n=177)	MetS+ (n=127)	*P*-value
CD19+ B cells	340 [217;432]	368 [273;484]	374 [264;579]	0.001
Transitional B cells	16 [9;25]	17 [11;27]	16 [11;28]	0.744
Naïve B cells	170 [17;276]	158 [14;306]	184 [28;286]	0.037
Non-switched B cells	24 [5;52]	22 [3;46]	24 [5;47]	0.037
Switched B cells	39 [23;74]	48 [29;80]	37 [21;69]	0.037
Double negative B cells	16 [10;108]	26 [11;222]	19 [10;150]	0.037
Plasma blasts	12 [7;19]	16 [9;30]	15 [9;31]	< 0.001

MetS-, morbidly obese patients without metabolic syndrome; MetS+, morbidly obese patients with metabolic syndrome.

All absolute numbers are presented as median [interquartile range]. P-values are after correction for covariates using a binomial mixed regression model.

### Absolute Numbers of NK Cells Are Increased in Morbidly Obese Patients

Absolute numbers of NK cells were increased in the morbidly obese patients as compared to lean controls (*P*<0.001), as shown in **Table 2C**. These differences were not significantly affected by the confounders that we corrected for. There was no difference in absolute numbers of NK cells between the MetS- and MetS+ group (*P*=0.182). When comparing the two different phenotypes of the NK cells, there was an increase in absolute numbers of both cytotoxic (CD56^dim^) and immunomodulatory (CD56^high^) NK cells (*P*<0.001), **Figure 1D**. These differences were not seen when comparing the MetS- with the MetS+ groups (*P*=0.459). Proportions of the various subsets were not significantly different between the three study groups (*P*=0.089), as shown in **Figure 2D**.

**Table 2c:** Absolute numbers of the NK cell subsets.

	Lean controls (n=55)	MetS- (n=177)	MetS+ (n=127)	*P*-value
CD56+ NK cells	163 [90;264]	219 [171;313]	257 [182;360]	< 0.001
Immunomodulatory NK cells	9 [7;14]	11 [8;19]	12 [8;17]	< 0.001
Cytotoxic NK cells	147 [84;248]	200 [146;281]	234 [164;328]	< 0.001

MetS-, morbidly obese patients without metabolic syndrome; MetS+, morbidly obese patients with metabolic syndrome; NK, natural killer.

All absolute numbers are presented as median [interquartile range]. P-values are after correction for covariates using a binomial mixed regression model.

### Monocyte Phenotype Is Not Affected in Morbidly Obese Patients

Similarly to the T cells, B cells and NK cells, the absolute number of monocytes was increased in the morbidly obese patients as compared to the lean controls (*P*<0.001) **Table 2D**. This was, however, not significantly different between the MetS- and MetS+ groups (*P*=0.163). Whereas there was no phenotypic change in monocytes with respect to percentages (*P*=0.832), **Figure 2E**, there was a significant increase absolute number of all three monocyte subsets between the LC and morbidly obese patients (*P*=0.002), **Figure 1E**. However, there were no differences between the MetS- and MetS+ groups.

**Table 2d:** Absolute numbers of the monocyte subsets.

	Lean controls (n=55)	MetS- (n=177)	MetS+ (n=127)	*P*-value
CD14+ monocytes	313 [232;414]	392 [293;507]	423 [315;558]	< 0.001
Non-classical monocytes	14 [9;19]	18 [12;29]	18 [11;27]	0.002
Intermediate monocytes	14 [9;23]	26 [15;37]	21 [15;36]	0.002
Classical monocytes	248 [166;334]	312 [228;405]	343 [257;473]	0.002

MetS-, morbidly obese patients without metabolic syndrome; MetS+, morbidly obese patients with metabolic syndrome.

All absolute numbers are presented as median [interquartile range]. P-values are after correction for covariates using a binomial mixed regression model.

## Discussion

In this study, cells of both the adaptive as well as the innate immune system proved to be affected by morbid obesity. Whereas MetS only induced an increase in CD4+ T cells, the absolute number of CD3+ T cells was also increased in morbidly obese patients as compared to lean controls. This increase in CD3+ T cells was amplified by CMV seropositivity. Furthermore, the CD8+ T cell differentiation was enhanced in morbidly obese patients, which was not affected by MetS and CMV seropositivity. For the innate immune system, absolute numbers of both monocytes and NK cells were increased in morbidly obese patients. However, this was not significantly different between the MetS- and MetS+ groups. Additionally, neither morbid obesity or MetS induced a phenotypic change in the NK cell and monocyte subsets. CMV seropositivity did not influence these results.

Ageing is associated with changes in composition of immune cell subsets, including the loss of naive CD8+ T cells and an increase in differentiated CD8+ T cells (29). We found a differentiated composition of CD8+ T cells in the morbidly obese patients as compared with lean controls, which is comparable to the increase of differentiated CD8+ T cells in the aging population. In contrast to an aging population, there was no loss of naive and RTE CD8+ T cells in our study population (30). The increase in both CD8+ EMRA T in the morbidly obese patients is comparable to what has been described in literature, in which immunological changes due to obesity were most pronounced in the CD8+ T cells (24, 31). In this study, this accelerated differentiation of CD8+ T cells was not only associated with morbid obesity, but also with increasing age. The accelerated aging of CD8+ T cells was not influenced by CMV seropositivity, which is in contrast to what has been described in literature (32). It might be that this accelerated differentiation is not further enhanced by CMV.

In elderly populations, a more differentiated profile of B cells with an increase of DN B cells has been described (33). In a study performed by Frasca et al., an increase of late/exhausted memory B cells was described among young individuals with obesity as compared to elder individuals with obesity and lean young and elderly controls (23). In our study, a comparable increase of DN B cells was seen in both MetS- and MetS+ groups as compared to the lean controls.

In this study, we have found a specific differentiated profile of the adaptive immune system of CD8+ T cells and B cells in morbidly obese patients, suggesting that there is obesity induced metaflammation. This aging profile consisted of an increase in more differentiated immune cells, being CD8+ EMRA T cells and DN B cells, while the number of immature immune cells was similar between the three study groups. Therefore, our data suggests that the production of immature immune cells is not disturbed by obesity. However, obesity does induce accelerated differentiation of CD8+ T cells and B cells. In our previous study, we found that T cell aging is partially reversed after bariatric surgery (24). Thus a long-term follow-up study in morbidly obese patients who will undergo bariatric surgery is suggested, in order determine whether excessive weight loss can reverse the aged composition of the different immune cell subsets.

An increased age is associated with an inverted CD4/CD8 ratio (34). In contrast to this, we found an increase in the CD4/CD8 ratio in the morbid obese patients, which confirms earlier studies in obesity (35, 36). The increase in CD4/CD8 ratio is especially explained by the increase in total CD4+ T cell numbers, whereas the CD8+ T cell numbers remains comparable between obese and lean subjects.

Despite the increase in absolute numbers of NK cells and monocytes, both cell types did not show an aged subset profile. Literature reports contradicting results on NK cell subset composition. Some studies show an increase in cytotoxic NK cells in morbidly obese patients, whereas other studies show an increase in immunomodulatory NK cells (19, 21, 37). These studies consisted of study populations of less than 20 patients. In our study, which was performed in a large study cohort, there was an increase of both cytotoxic and immunomodulatory NK cells. However, no obesity-induced senescence of NK cells was found.

We found an increase in CD14^dim^ monocytes in morbidly obese patients that what comparable to what has been observed previously (38, 39). Similarly to Poitou et al., this increase was seen in morbidly obese patients as compared to lean controls, but was not seen in MetS- as compared to MetS+. Thus, MetS does not seem to influence aging of monocytes. Poitou et al. describes a decrease of CD14^dim^ monocytes after LRYGB, suggesting that the monocyte aging is reversible. However, that study group only consisted of 36 patients, and it would therefore be interesting to duplicate this study in a larger cohort.

Although, to our knowledge, this is the first large and comprehensive study investigating immunosenescence in morbidly obese patients, a limitation of this study is that we have only focused on the composition of the immune system. Previous studies have shown an increase in proinflammatory and a decrease of anti-inflammatory cytokine production in morbidly obese individuals, which causes DNA damage and is associated with age-related diseases and mortality (29, 40). Therefore a study to the functioning of the immune system in this large population of morbidly obese patients as compared to lean controls is recommended.

Oxidative and nitrosative stress play an important role in the development of metabolic diseases (41). Increased levels of serum myeloperoxidase were previously observed in patients with metabolic syndrome, indicating that inflammation is intensified in this patient population (42). Glutathione deficiency gives oxidative stress, leading to an accelerated aging and diseases such as T2D. Glutathione oxidation was increased in patients with obesity and hypertension, but not specifically in patients with metabolic syndrome (42). Bariatric surgery reduces protein glycoxidation and nitrosative stress (43). It would be interesting to also identify the markers on our large study cohort, and additionally assess these markers in the cohort after bariatric surgery.

In conclusion, obesity-induced effects on the composition of the immune system are confined to shifting of the CD8+ T cell and B cell compartment to a more differentiated phenotype. Further research is required to evaluate whether bariatric surgery reverses this differentiated phenotype, as well as research into the function of the immune cells of morbidly obese individuals, both before and after bariatric surgery.

## Data Availability Statement

The raw data supporting the conclusions of this article will be made available by the authors, without undue reservation.

## Ethics Statement

The studies involving human participants were reviewed and approved by Medical Research Ethics Committees United (MEC-U). The patients/participants provided their written informed consent to participate in this study.

## Author Contributions 

LW, EH, RK, MD, RB, and NL contributed to the design and implementation of the research. LW, GA, and MK carried out the experiments. LW and TK performed the analysis of the results. LW took the lead in writing the manuscript in consultation with RB and NL. All authors contributed to the article and approved the submitted version.

## Funding

The materials for immune cell phenotyping were funded by Medtronic, Netherlands. The publication fee was funded by the Maasstad Hospital Science Fund.

## Conflict of Interest

The authors declare that the research was conducted in the absence of any commercial or financial relationships that could be construed as a potential conflict of interest.
