# Supplementary material for: Effects of Morbid Obesity and Metabolic Syndrome on the Composition of Circulating Immune Subsets
Source: Front Immunol. 2021 Jul 20;12:675018. doi: 10.3389/fimmu.2021.675018 (PMC8330422; doi:10.3389/fimmu.2021.675018)
Supplement: Supplementary file 1 [file Table_1.pdf]

SUPPLEMENTARY TABLE 1 | Immune cells and their corresponding markers

Immune cell	Marker
T cell subsets	
Recent thymic emigrants	$CD31^{+}$ $CD45RO^{-}CCR7^{+}$
Naive T cells	$CD45RO^{-}CCR7^{+}$
Central memory T cells	$CD45RO^{+}CCR7^{+}$
Effector memory T cells	$CD45RO^{+}CCR7^{-}$
Terminally differentiated effector memory T cells	$CD45RO^{-}CCR7^{-}$
Advanced differentiated T cells	$CD28^{null}$
NK T cell subset	
Immunomodulatory NK cells	$CD56^{bright}CD16^{-}$
Cytotoxic NK cells	$CD56^{dim}CD16^{+}$
B cell subsets	
Naive B cells	$CD27^{-}IgD^{+}$
Non-switched B cells	$CD27^{+}IgD^{+}$
Switched B cells	$CD27^{+}IgD^{-}$
Double-negative B cells	$CD27^{-}$IgD-
Transitional B cells	$CD24^{high}CD38^{ihgh}$
Plasma blasts	$CD27^{high}CD38^{high}$
Monocyte subset	
Classical monocytes	$CD14^{+}CD16^{-}$
Non-classical monocytes	$CD14^{+}CD16^{+}$
Intermediate monocytes	$CD14^{dim}CD16^{+}$

NK, natural killer

SUPPLEMENTARY FIGURE 1 | Flowcytometry gating strategies

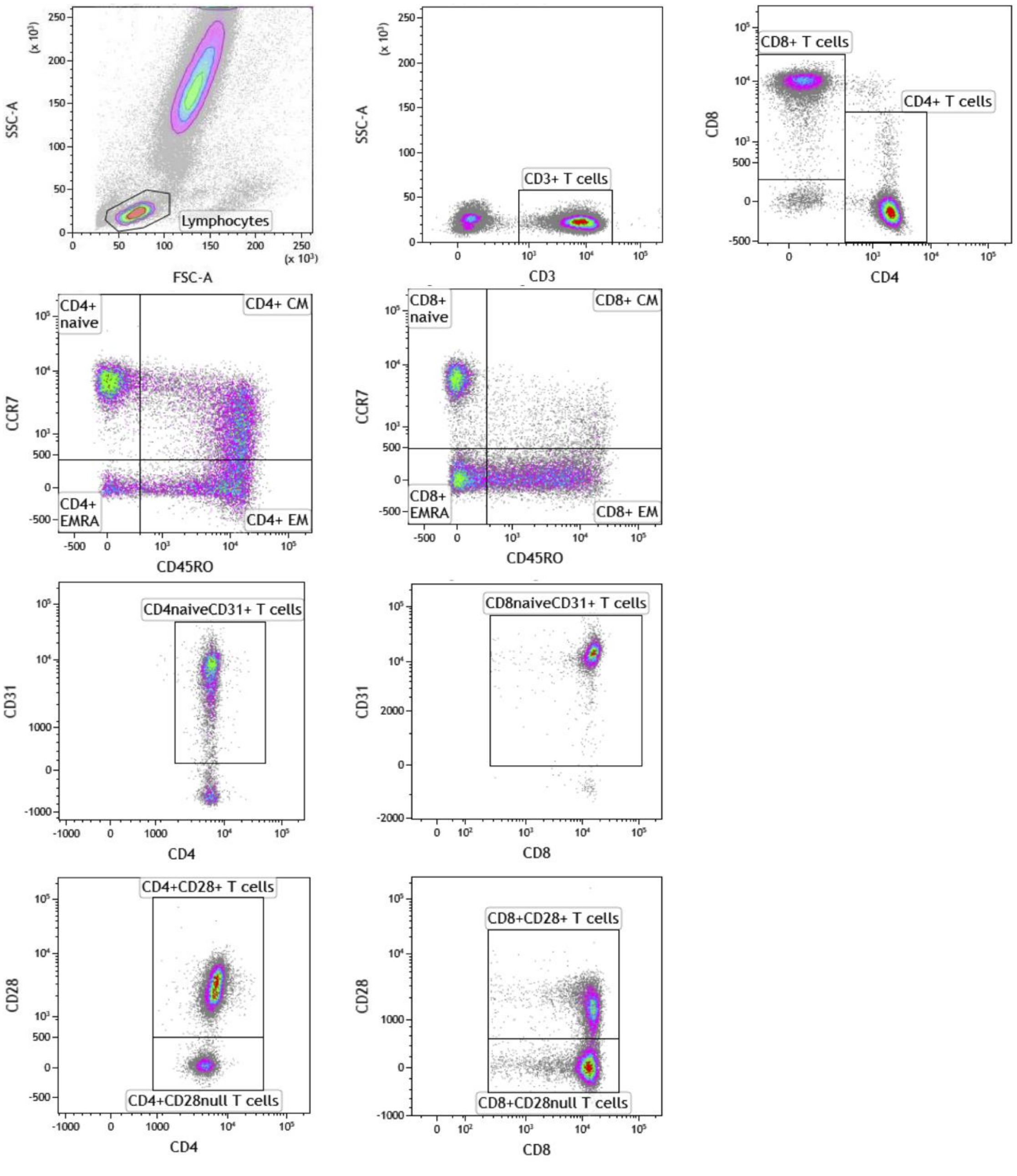

A. Flow cytometry gating strategy for CD4+ and CD8+ T cell subsets

Lymphocytes were gated and then viable CD3+ T cells were selected. These were then selected for either CD4+ or CD8+ T cells. Afterwards, the CD4+ and CD8+ T cells were subdivided into the main T cell subsets using CCR7 and CD45RO into $CD45RO^{-}CCR7^{+}$ naive, $CD45RO^{+}CCR7^{+}$ CM, $CD45RO^{+}CCR7^{-}$ EM and $CD45RO^{-}CCR7^{-}$ EMRA T cells. Within naive T cells, $CD31^{+}$ RTEs were then selected. Additionally, CD4+ and CD8+ T cells were plotted against CD28 and the $CD28^{null}$ T cells were gated.

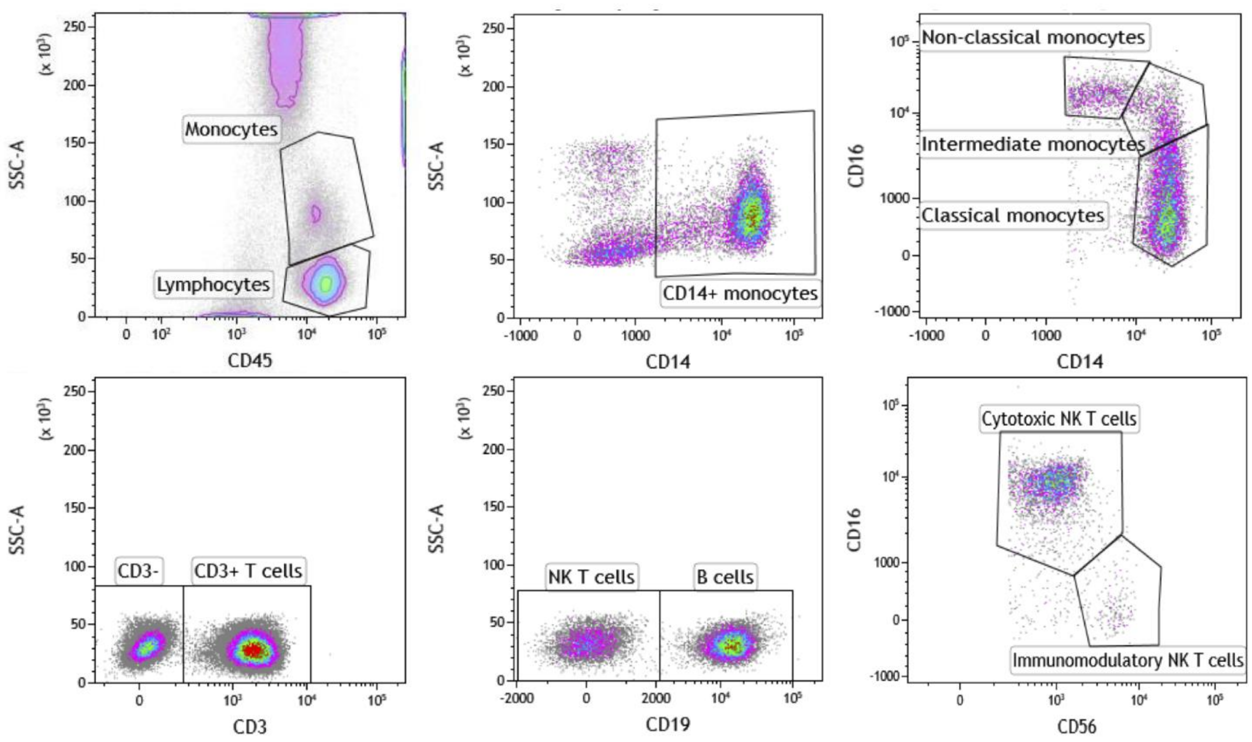

B. Flow cytometry gating strategy for NK cell and monocyte subsets

First, the monocytes were gated using SSC and CD45. Then, monocytes were selected using CD14 and afterwards the monocytes were subdivided into CD14^{dim}CD16^{bright} non-classical, CD14^{+}CD16+ intermediate and CD14^{+}CD16^{-} classical monocytes.

Second, the viable lymphocytes were gated and then the CD3^{-} cells were selected. These cells were plotted against CD19 and CD19^{-} NK cells were then gated. Afterwards, the NK cells were subdivided into CD56^{dim}CD16^{+} cytotoxic and CD56^{bright}CD16^{-} immunomodulatory NK cells.

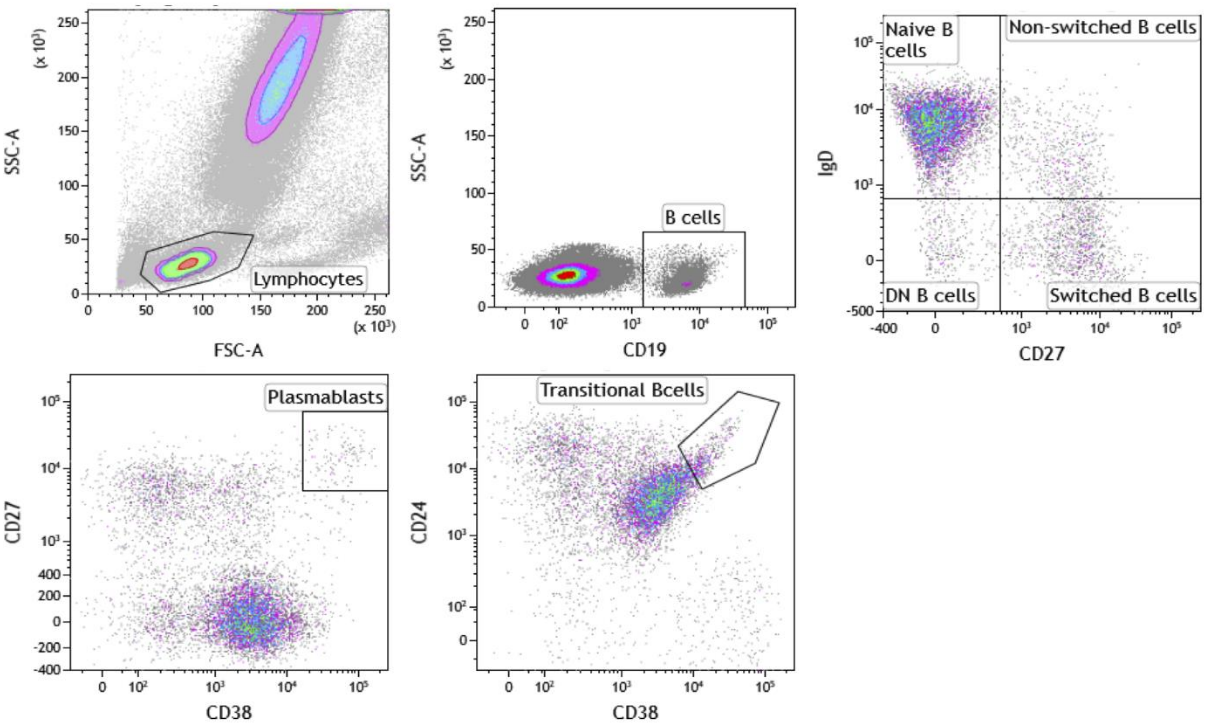

C. Flow cytometry gating strategy for B cell subsets

Viable lymphocytes were gated and then plotted against CD19. $CD19^+$ B cells were then gated and subdivided into the main B cell subsets using CD27 and IgD into $CD27^-IgD^+$ naive, $CD27^+IgD^+$ non-switched, $CD27^+IgD^-$ switched and $CD27^-IgD^-$ double negative B cells. Additionally, the B cells were plotted against CD27 and CD38 and $CD27^{high}CD38^{high}$ plasmablasts were gated. At last, the B cells were plotted against CD24 and CD38 and the $CD24^{high}CD38^{high}$ transitional B cells were gated.
